# The Role of Bcl-xL Protein in Viral Infections

**DOI:** 10.3390/ijms22041956

**Published:** 2021-02-16

**Authors:** Zbigniew Wyżewski, Weronika Świtlik, Matylda Barbara Mielcarska, Karolina Paulina Gregorczyk-Zboroch

**Affiliations:** 1 Institute of Biological Sciences, Cardinal Stefan Wyszyński University in Warsaw, 01-815 Warsaw, Poland; 2 Department of Biochemistry and Microbiology, Institute of Biology, Warsaw University of Life Sciences-SGGW, 02-787 Warsaw, Poland; weronika_switlik@sggw.edu.pl; 3 Institute of Veterinary Medicine, Warsaw University of Life Sciences, 02-787 Warsaw, Poland; matylda_mielcarska@sggw.edu.pl (M.B.M.); karolina_gregorczyk_zboroch@sggw.edu.pl (K.P.G.-Z.)

**Keywords:** Bcl-xL, HBV, HCV, HIV, IAV, EBV, HTLV-1, MRBV, SBV, CoV

## Abstract

Bcl-xL represents a family of proteins responsible for the regulation of the intrinsic apoptosis pathway. Due to its anti-apoptotic activity, Bcl-xL co-determines the viability of various virally infected cells. Their survival may determine the effectiveness of viral replication and spread, dynamics of systemic infection, and viral pathogenesis. In this paper, we have reviewed the role of Bcl-xL in the context of host infection by eight different RNA and DNA viruses: hepatitis B virus (HBV), hepatitis C virus (HCV), human immunodeficiency virus (HIV), influenza A virus (IAV), Epstein-Barr virus (EBV), human T-lymphotropic virus type-1 (HTLV-1), Maraba virus (MRBV), Schmallenberg virus (SBV) and coronavirus (CoV). We have described an influence of viral infection on the intracellular level of Bcl-xL and discussed the impact of Bcl-xL-dependent cell survival control on infection-accompanying pathogenic events such as tissue damage or oncogenesis. We have also presented anti-viral treatment strategies based on the pharmacological regulation of Bcl-xL expression or activity.

## 1. Introduction

Bcl-extra-large (Bcl-xL; UniProtKB: Q07817) is a member of the Bcl-2 protein family, a group of regulatory molecules involved in intrinsic apoptosis pathway regulation. The Bcl-2 family consists of both pro-apoptotic and anti-apoptotic factors. The first group is represented by three BH domains (i.e., Bax and Bak) as well as BH3-only proteins (i.e., Bim, Bad, Bid, Bik, Bmf, and Hrk). The anti-apoptotic Bcl-2 family members comprise a protein with four BH domains, including Bcl-2, Bcl-w, A1, Mcl-1, and Bcl-xL [1,2,3,4]. 

The effect of Bcl-xL activity on the apoptotic cell potential has been widely studied in the areas of tumor development [5,6]. Indeed, cell viability can be a potent factor to determine various diseases. Upregulation of the intracellular level of Bcl-xL is associated with the development of tumors, such as prostate cancer [7], lymphomas [8], advanced malignant melanoma [9], neuroblastoma [10], breast cancer [11], and many others. Bcl-xL also seems to be an important player in viral infections. In this paper, we have reviewed reports presenting the role of Bcl-xL in infections caused by different viral pathogens: hepatitis B virus (HBV), hepatitis C virus (HCV), human immunodeficiency virus (HIV), influenza A virus (IAV), Epstein-Barr virus (EBV), human T-lymphotropic virus type-1 (HTLV-1), Maraba virus (MRBV), Schmallenberg virus (SBV) and coronavirus (CoV).

## 2. The Role of Bcl-xL and Other Bcl-2 Family Members in the Regulation of Apoptosis

Apoptosis may be induced via two different cascades of signal transduction, the extrinsic and intrinsic pathway. The first one requires extracellular ligands to activate the cell surface receptors of the tumor necrosis factor (TNF) superfamily (TNFSF) [12]. For example, the first apoptosis signal (Fas) ligand (FasL) and TNF-α can stimulate Fas protein and TNFR1, respectively [13,14]. Activation of TNFSF members initiates intracellular signal transduction, and the cell undergoes apoptosis via a caspase-8/-3-mediated mechanism [15]. The intrinsic pathway is strictly dependent on the integrity and functionality of mitochondria as its essential property is to release pro-apoptotic markers (i.e., cytochrome c) from the mitochondrial intermembrane space (IMS) to the cytosol [16,17]. As a result, it increases the outer mitochondrial membrane (OMM) permeability. The unsealing of mitochondria and the consecutive caspase activation cascade leads to programmed cell death [18,19,20].

Permeabilization of the OMM consistently results in the mitochondrial permeability transition (MPT). The MPT is a process that opens non-specific permeability transition pores (PTPs) located in the inner mitochondrial membrane (IMM) to communicate with the OMM. The PTP is a protein complex composed of a voltage-dependent anion channel (VDAC), benzodiazepine peripheral receptor (BPR), and an adenine nucleotide translocator (ANT) [21,22,23,24]. Under normal circumstances, the MPT appears in a small number of mitochondria without any substantial influence on the mitochondrial membrane potential (ΔΨm). Pro-apoptotic stimulation increases the MPT frequency, resulting in the loss of ΔΨm, permeabilization of the OMM, and the release of mitochondrial proteins to the cytosol [25]. The Bcl-2 protein family members can be recruited to the PTP and contribute to MPT regulation. Bax and Bak indirectly interact with the VDAC, resulting in the increase in PTP diameter until the release of cytochrome c, whereas Bcl-xL is a negative regulator of the mechanism [26].

**Figure 1:**
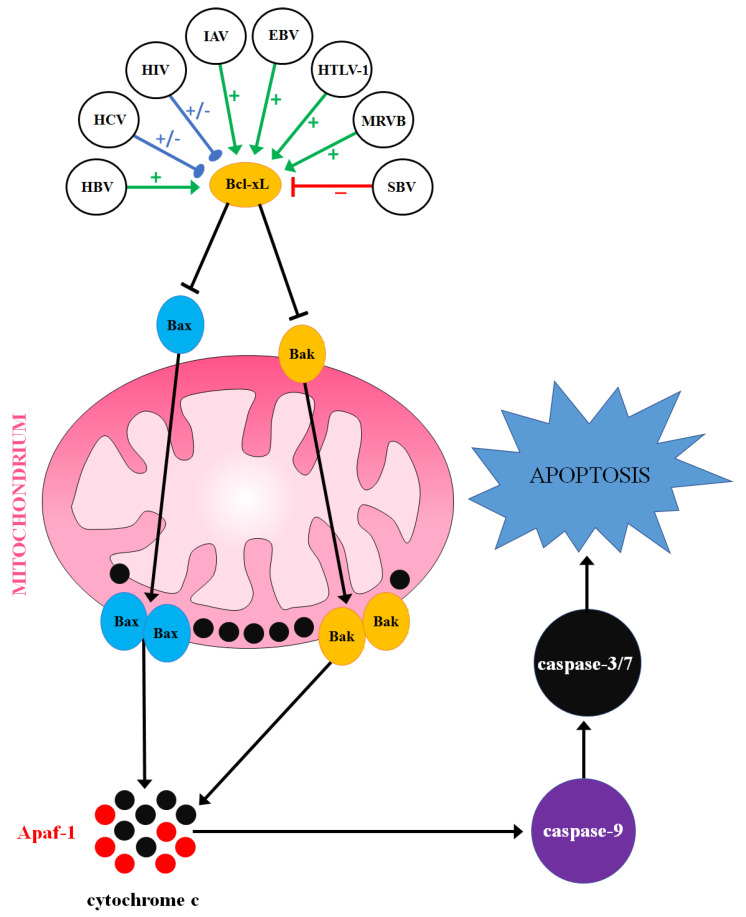
The influence of viral infections on the cell viability *via* regulation of Bcl-extra-large (Bcl-xL) expression. The color of arrows represents the influence of viral infections on Bcl-xL expression (green: upregulation; red: downregulation; blue: upregulation or downregulation, according to the circumstances).

Another inherent operation of the intrinsic apoptosis induction is the PTP-independent oligomerization of the pro-apoptotic Bcl-2 family members. The group consists of proteins that interact with each other to promote a mitochondrial pathway of type I programmed cell death, or to counteract it, affecting mitochondrial integrity [25,26,27]. Active forms of the three domain members of the Bcl-2 protein family, Bax and Bak, are able to integrate into the OMM, oligomerize within a lipid bilayer, and form pores that enable cytochrome c and other pro-apoptotic factors to escape the intermembrane mitochondrial space and move to the cytosol. In the cytosol, cytochrome c binds apoptotic protease activating factor-1 (Apaf-1) to form an apoptosome, a complex responsible for the initiation of the caspase cascade via caspase-9 activation. The anti-apoptotic Bcl-2 family proteins, such as Bcl-xL, can bind Bax and Bak to inactivate them and prevent PTP-independent mitochondrial permeabilization [28,29,30]. Many viruses are able to upregulate or downregulate Bcl-xL to affect the viability of the cell (Figure 1).

## 3. The Role of Bcl-xL in HBV Infection

Hepatitis B virus (HBV) is a member of the *Hepadnaviridae* family [31] and the *Orthohepadnavirus* genus [32]. Its genome has the form of partially double-stranded DNA that undergoes reverse transcription during infection. Genetic material is enclosed in an icosahedral capsid, encircled by a lipoprotein envelope [33,34]. The diameter of infectious viral particles is 44 nm [35]. The DNA genome comprises four open reading frames that overlap each other and encode seven proteins: polymerase (pol), a structural capsid protein named HBV core antigen (HBcAg), HBV x protein (HBx), secreted HBV e antigen (HBeAg), and three forms of surface proteins (HBsAg): large (L), medium (M), and small (S) ones [36,37,38]. Studies on HBV are very important, because a combination of pathogenicity, genome variability, and high prevalence of the virus may pose serious problems for modern medicine. HBV is an etiological agent of both acute and chronic hepatitis [36]. Viral infection may lead to liver cirrhosis and hepatocellular carcinoma (HCC) [39]. Deficiency in the proofreading activity of HBV reverse transcriptase causes high mutation rates of the viral genome, resulting in the emergence of ten HBV genotypes with different geographic localization [39,40,41]. It is estimated that approximately 257 million people worldwide are infected with HBV [36].

Research has shown that HBV-host interactions included contact between HBx and anti-apoptotic members of the Bcl-2 family. HBx targets Bcl-xL and Bcl-2 via its BH3-like motif. Geng et al. [42] have determined that interaction between HBx and Bcl-2 family proteins has a significant impact on viral pathogenesis. During the experiment, the mutations in the BH3-like motif deprived HBx of the ability to bind Bcl-xL and Bcl-2, abolishing the HBx-dependent increase in the cytosolic calcium level in infected hepatocytes. Mutational inactivation of HBx prevented cell death and markedly reduced viral replication, a process requiring high levels of cytosolic Ca^2+^. Silencing Bcl-xL expression with the use of short hairpin RNA (shRNA) has shown the importance of anti-apoptotic protein in viral replication. Bcl-xL knockdown reduced the cytosolic calcium level in the infected HepG2 cells, decreasing HBV replication. Summarizing, the obtained results have suggested that Bcl-xL cooperated with HBx to promote viral reproduction and infected cell death [42]. Zhang et al. have analyzed the crystal structure of the HBx BH-3-like motif bound to Bcl-xL protein. Exploring the motif further, the team has identified a short α-helix and its ability to enter the hydrophobic pocket of Bcl-xL [43]. Because of its role in the HBV-host cell interaction, Bcl-xL should be considered an antiviral treatment [42,43].

Another reason behind examining Bcl-xL further is its association with hepatocarcinogenesis. Upregulation of Bcl-xL expression causes a decrease in the apoptotic potential of HCC cells that contributes to their survival, intensified growth, and colony formation [44]. The study of Kong et al. [45] on HBV-positive HCC has shown that HBV causes an increase in Bcl-xL in an IL-34-dependent manner [45].

## 4. The Role of Bcl-xL in HCV Infection

Hepatitis C virus (HCV) belongs to the *Flaviviridae* family [46] and the *Hepacivirus* genus [47]. Its genome has the form of positive-sense, single-stranded RNA (ssRNA), and is enclosed in an icosahedral capsid. The enveloped virion is 50–60 nm in diameter. The RNA genome contains one open reading frame [48] that is flanked by two non-coding regions (5’-NTR and 3’-NTR) and encodes one viral polyprotein. After expression, the product is cleaved into the structural proteins (i.e., protein capsid and two envelope proteins) and the non-structural (NS) ones, including proteases and viral RNA-dependent RNA polymerase (vRdRp) [49,50]. HCV is the etiological agent of chronic liver disease that can develop into cirrhosis. HCV infection may also lead to hepatocellular carcinoma [51]. It is estimated that 170 million people worldwide are chronically infected with HCV [52].

Scientific studies have suggested that the downregulation of Bcl-xL may play an important role in intracellular HCV infection and pathogenesis [53]. Scientific findings suggest that the pathogenic processes promote the death of infected cells. Apart from necrosis and autophagy, hepatocyte apoptosis plays a significant role in the development of liver disease [54]. Javed and Manzoor [53] have examined levels of two Bcl-2 family members, anti-apoptotic Bcl-xL and pro-apoptotic Bax, in Huh-7 cells (hepatocytes derived from the cellular carcinoma cell line) transfected with NS4A and NS3-NS4A of HCV genotype 3a. The presence of viral proteins resulted in a decrease in the intracellular level of Bcl-xL and a parallel promotion of Bax expression. A substantial change in the proportion Bcl-xL/Bax led to a series of pro-apoptotic molecular events, including Bax translocation to the mitochondria and the initiation of a caspase cascade preceding cell death [53]. In another study, Joyce et al. [55] observed damage to and the apoptosis of human hepatocytes in chimeric mice (animals transplanted with human liver cells) inoculated with HCV. The viral infection led to an increase in total Bcl-xL levels in the liver, however, within HCV-positive organs, Bcl-xL expression in the HCV-infected cells was lower than in the non-infected ones. The results suggested that hepatocytes responded to the liver infection-associated stress by upregulation of Bcl-xL and promoting their survival, whereas HCV impaired this response by counteracting the increase in Bcl-xL and favoring apoptosis [55]. 

Pharmacological regulation of hepatocyte apoptosis is considered a promising therapeutic strategy against HCV. Yang et al. [56] have used an animal model of HCV infection, *Tupaia belangeri*, to examine the efficacy of xanthohumol, a prenylated chalcone isolated from hops, in liver disease treatment. The therapeutic agent had a beneficial influence on the HCV-infected animals, which was manifested by a reduction of steatosis, fibrosis, and hepatic inflammation. The findings of the team enabled them to determine the possible association between liver condition improvement and the decrease in the apoptotic potential of hepatocytes. Xanthohumol exerted its therapeutic effect by increasing the expression of Bcl-xL and lowering the level of Bax in the liver tissues. Therefore, the Bcl-xL was shown to be a factor that might affect the clinical picture of liver pathogenesis by participating in the regulation of hepatocyte apoptosis and the enhancement of Bcl-xL’s contribution to the treatment effect by promoting cell survival [56].

On the other hand, Bcl-xL may also be co-responsible for HCV-induced oncogenesis. Research by Guo et al. [57] on human fetal liver stem cells (hFLSCs) infected with HCV has revealed nuclear factor-ĸB (NF-ĸB)-dependent mechanisms that counteract HCV-induced apoptosis at the late stage of infection. One of the cell survival-promoting events was the upregulation of intracellular Bcl-xL [57]. Meanwhile, previous studies had found the overexpression of Bcl-xL in HCCs and suggested the contribution of its anti-apoptotic activity to HCC survival under various stress conditions, and to hepatocellular carcinoma development [58,59].

## 5. The Role of Bcl-xL in HIV Infection

Human immunodeficiency virus (HIV) belong to the *Retroviridae* family and the *Lentivirus* genus [60]. Its genome has the form of two copies of identical, positive-sense, single-stranded RNA, each of them containing a complete set of genetic information necessary for HIV to replicate in the host cell. An essential step in viral reproduction is reverse transcription that is indispensable in the production of the double-stranded DNA (dsDNA) molecule that integrates into the host genome [60,61]. Genetic material is encased in an enveloped, cone-shaped capsid [62]. An average mature virion diameter is 145 nm [63]. The HIV DNA genome comprises several genes located between long terminal repeats (5’-LTR and 3’-LTR) at either end. Among the HIV genes, *gag*, *pol*, and *env* encode precursors of inner structural proteins, viral enzymes (including reverse transcriptase), and envelope proteins, respectively. The *env* open reading frame is followed by the *nef* gene encoding multifunctional negative regulating factor (Nef). Another important HIV gene is *vpr* and its product is pro-apoptotic viral protein R (Vpr) [60]. HIV is the etiological agent of acquired immunodeficiency syndrome (AIDS) and remains one of the major challenges for modern medicine. It poses a serious public health problem due to its ability to hide inside the immune cells of an HIV-positive individual [64]. Estimates show that the number of HIV-1-positive people is approximately 38 million individuals worldwide [65]. The HIV pandemic has spread across the whole world and, at present, there is no region free from the virus and the disease it causes [64].

An early study of Petrovas et al. [66] examined the influence of HIV infection on the Bcl-xL level in HIV-specific CD8^+^ T lymphocytes [66], host cells responsible for mediating the immune response against the pathogen [67]. The team found an HIV-dependent decrease in the Bcl-xL expression, a phenomenon that, along with Bcl-2 deficiency, seemed to elevate cell sensitivity to apoptosis. To confirm the potent role of the anti-apoptotic Bcl-2 family members in maintaining CD8^+^ T cell viability, IL-15, a cytokine promoting their activation and proliferation, was implemented. HIV-infected CD8+ T cell cultures treated with IL-15 were resistant to apoptosis, and their survival coincided with the stimulation of Bcl-xL and Bcl-2 expression. The study indicated the contribution of the anti-apoptotic Bcl-2 protein family to the antiviral effect of the IL-15 treatment [66].

HIV infection is extremely difficult to treat because of the virus’s ability to hide inside immune cells. The cellular reservoirs consist of memory CD4^+^ T lymphocytes and myeloid cells including macrophages. Those cells constitute a “refuge” responsible for the persistence of the viral infection. Therefore, the ability of infected macrophages to counteract apoptosis promotes the spread of the virus [68]. Meanwhile, previous studies of Sanz et al. [69] have shown that the differentiation of hematopoietic progenitor cells in macrophages is associated with a substantial increase in the intracellular level of Bcl-xL [69]. Research by Choi and Smithgall [70] has revealed that the viral Nef protein enhances the expression of Bcl-xL in a human macrophage precursor cell line, TF-1. Nef-dependent upregulation of Bcl-xL via the Erk signaling pathway promoted cell resistance to apoptotic stimulation by cytokine deprivation. Further analysis by Busca et al. [71] has elucidated that Bcl-xL (together with another anti-apoptotic representative of the Bcl-2 family, Mcl-1) determines the overall cell viability, as the silenced expression of Bcl-xL and Mcl-1 results in macrophage apoptosis. However, the team revealed that resistance to Vpr-induced cell death was achieved via a Bcl-xL-independent mechanism [71].

Many reports are devoted to the mechanisms of AIDS-associated disorders. HIV infection may lead to neurocognitive impairment. It can replicate in the immune system and supporting cells of the central nervous system (CNS) to provoke an immune–inflammatory response [72,73]. HIV-positive macrophages release neurotoxins that cause the death of host neuronal cells [74]. Chen et al. [74] have studied the potential role of Bcl-xL in the cellular response to HIV infection. The team used NT2.N/Bcl-xL neuronal cell lines characterized by the overexpression of Bcl-xL to determine whether the anti-apoptotic Bcl-2 family members are able to protect neurons from HIV-1-infected macrophages. The experiment showed that high levels of Bcl-xL result in resistance to the intrinsic apoptosis pathway induced by macrophage neurotoxins. The results suggest considering Bcl-xL overexpression in neuronal cells as part of a strategy for the treatment of AIDS-associated disorders [74].

## 6. The Role of Bcl-xL in Influenza A Virus Infection

Influenza A virus (IAV) is a viral genus representing the *Orthomyxoviridae* family [75]. The IAV genome has the form of negative-sense ssRNA divided into eight segments and enclosed in an enveloped virion [76] of 100 nm in diameter [77]. Each segment encodes at least one viral protein. Segments 1, 2, and 3 consist of genes for PB2, PB1, and PA proteins, respectively, and are subunits of vRdRP. Segments 4 and 6 encode hemagglutinin (HA) and neuraminidase (NA), respectively, and are both located on the surface of the virion envelope. The IAV serotypes are dependent on the variants of HA and NA [76]. IAV infects animals such as birds [78], seals [79], ferrets [80], mink [81], pigs [82], horses, and dogs, but also poses a threat to humans as a zoonotic agent. The main reservoir for IAV is aquatic birds [83]. IAV is often responsible for mild infections in humans. However, a few serotypes, such as H1N1, H5N1, H5N6, H10N8, and H7N9, have proven to be the cause of severe illnesses or even death in infected patients [84].

Research by Kakkola et al. [85] has indicated that the anti-apoptotic Bcl-2 family members may be involved in effective viral replication at the early stage of infection. The team has revealed that, in humans, in IAV-infected retinal pigment epithelium (RPE) cells, the interaction of Bcl-xL, Bcl-2, and Bcl-w with Bax, Bad, and the uveal autoantigen with coiled-coil domains and ankyrin repeats (UACA) counteracted the OMM permeabilization, thwarted the consequent release of pro-apoptotic factors from the mitochondria to the cytosol, and prevented the downstream signaling cascade. The role of Bcl-xL, Bcl-2, and Bcl-w seemed to be to delay the intrinsic apoptosis induction at the same time as maintaining cell viability at the early stage of infection to enable the synthesis of progeny viral ribonucleoprotein (vRNP) in the nucleus. The report of Kakkola et al. [85] suggests improving the anti-IAV therapies by diminishing the activity or reducing the intracellular level of the anti-apoptotic Bcl-2 family members, including Bcl-xL. The team has examined the pro-apoptotic activity of the anti-cancer compound ABT-263 in IAV-infected cells. In light of the proposed model, at the early stage of the infection, ABT-263 binds Bcl-xL and enhances its dissociation from Bax, Bad, and UACA. After release from the inactivating complex with Bcl-xL, Bax and Bad cause mitochondria permeabilization and start the intrinsic apoptosis pathway (with free UACA as one of the downstream pro-apoptotic molecular players). Therefore, the ABT-263–Bcl-xL interaction impairs the metabolism and functions of an infected cell and thus interrupts viral replication. The obtained results have suggested the possible effectiveness of treatment strategies that use Bcl-xL as a potential drug target [85].

On the other hand, the report of Lee et al. [86] has suggested that Bcl-xL may counteract virus spread by directing the cellular signaling pathways to pyroptosis. The team studied the possible role of Bcl-xL in the response of respiratory epithelial cells to IAV infection. The obtained results suggested that viral infection led to the substantial upregulation of Bcl-xL via Janus kinase/signal transducers and the activators of transcription (JAK-STAT) signaling pathway, a molecular cascade induced by type I interferon (IFN). In light of the report, it seems that the anti-apoptotic activity of Bcl-xL contributed to the switch of cellular signaling from the apoptotic program to the pyroptotic one at the late stage of IAV infection. The pyroptosis of infected respiratory epithelial cells induced an inflammatory response, leading to the mobilization of macrophages and neutrophils to eliminate the infected cells and stop the viral spread [86].

## 7. The Role of Bcl-xL in EBV Virus Infection

Epstein–Barr virus (EBV) is a member of the *Herpesviridae* family [87] and the *Lymphocryptovirus* genus [88]. Its genome has the form of linear double-stranded DNA [89,90] and is encased in an enveloped [91] icosahedral capsid [92]. The EBV virion is approximately 115 nm in diameter [93]. The viral DNA encodes over 85 genes, including DNA polymerase [94]. EBV is characterized by its very high prevalence—it is estimated that over 90% of the human population is persistently infected with the virus. The primary infection of EBV in infants does not occur with any specific symptoms—it can even be completely asymptomatic [88]. The virus is able to persist in some infected B lymphocytes and cause reactive lymphoid hyperplasia in immunodeficient hosts [95]. EBV-positive adults may also suffer from B cell lymphoproliferative disorders and lymphomas (i.e., Burkitt’s lymphoma) [96,97], undifferentiated nasopharyngeal carcinoma (NPC) [98], gastric carcinoma [99], chronic active EBV (CAEBV), or extranodal natural killer (NK)/T cell lymphoma (ENKTL) [100].

In light of scientific reports, it seems that Bcl-xL plays an important role in EBV-associated diseases. Portis et. al. [101] have revealed that one of the viral late gene expression products, latent membrane protein-2A (LMP-2A), is responsible for the upregulation of Bcl-xL expression in EBV-infected B lymphocytes, favoring cell viability and latent EBV infection, possibly contributing to EBV-associated tumorigenesis [101,102]. The study of Zhang et al. [103] has suggested that the EBV protein, BARF1, promoted gastric carcinoma development by inducing Bcl-xL and Bcl-2 expression in a process involving the mitogen-activated protein kinase (MAPK)/c-Jun signaling pathway [103]. Sejic et al. [100] have performed studies on cancer cell lines derived from individuals with CAEBV or the ENKTL. All of the tested tumor cells were resistant to DNA damage-inducing chemotherapeutic agents. Meanwhile, A-1331852, a chemical compound mimicking the BH-3 domain of Bcl-xL, was able to induce apoptosis. The results have suggested that Bcl-xL activity was responsible for CAEBV- and ENKTL-derived tumor cell viability and their sustained growth [100].

## 8. The Role of Bcl-xL in HTLV-1 Virus Infection

Human T-lymphotropic virus type-1 (HTLV-1) is a member of the *Retroviridae* family [104] and the *Deltaretrovirus* genus [105]. It contains two strands of positive-sense, single-stranded RNA. An enveloped virion is approximately 100 nm in diameter. During the infection, viral RNA undergoes reverse transcription, leading to the synthesis of double-stranded DNA that integrates into the host genome [106]. HTLV-1 DNA contains genes for structural and functional proteins (i.e., capsid protein, protease, polymerase, and envelope protein), and also comprises four overlapping open reading frames encoding regulatory factors [106,107]. HTLV-1 infects immune system cells, such as CD4^+^ and CD8^+^ T lymphocytes and monocytes. The infection tends to be asymptomatic, however, in 2–5% of infected people, it is associated with adult T cell leukemia/lymphoma (ATLL) or HTLV-1-associated myelopathy (HAM)/tropical spastic paraparesis (TSP) [107].

ATLL development is driven by the clonal expansion of CD4+CD25+ T cells, therefore, their viability seems to be a key factor determining the progress of the disease [108]. Studies have revealed that the transactivator from the X-gene region (Tax), a protein encoded by HTLC-1, is responsible for the upregulation of Bcl-xL levels in ATLL T lymphocytes [109,110]. Zhang et al. [108] have proposed Bcl-xL as the target of antiviral therapy. The team has examined navitoclax, an inhibitor of Bcl-xL activity. The tested agent, used in combination with the JAK inhibitor ruxolitinib, exerted a strong, anti-tumor effect in ATLL treatment while, acting alone, it showed modest therapeutic effectiveness. In ATLL cells, navitoclax combined with ruxolitinib caused pro-apoptotic events, including caspase-3/7 activation. An experiment on tumor-bearing mice showed that a reduction in cancer cell viability led to the inhibition of tumor growth and promoted survival of the tested animals [108]. Mori et al. [111] have determined the ability of rottlerin, a protein kinase C (PKC)-δ inhibitor, to kill HTLV-1 infected cells. The research showed that rottlerin prevented PKC-δ phosphorylation and caused several events that significantly decreased cell viability. One of them was the downregulation of Bcl-xL [111]. Another study on ATLL treatment strategies was devoted to the evaluation of cerdulatinib, a chemical agent inhibiting JAK and spleen tyrosine kinase (SYK). Ishikawa et al. [112] have determined its anti-proliferative and pro-apoptotic impact on HTLV-1 infected T lymphocytes. The activity of cerdulatinib led to a number of pro-apoptotic events, including a decrease in the intracellular level of Bcl-xL [112].

## 9. The Role of Bcl-xL in Maraba Virus Infection

Maraba virus (MRBV) is a member of the *Rhabdoviridae* family and the *Vesiculovirus* genus that contains a negative-sense ssRNA genome [113]. Its enveloped bullet-shaped virion is 170 nm in length and 70 nm in diameter. The viral genome comprises five open reading frames that encode a nucleocapsid protein, phosphoprotein, matrix protein, glycoprotein, and polymerase, respectively [114]. MRBV is closely related to vesicular stomatitis virus (VSV), an archetypical vesiculovirus that is well known for its oncolytic properties. The antigenic relationship with VSV makes MRBV a potential candidate in anti-tumor treatment [113,115]. Many scientific reports have shown that MRBV is non-pathogenic to humans, although its antigens have caused one documented case of seroconversion. Summarizing, the advantages presented above prove MRBV to be an effective and safe instrument in oncomedicine [114]. Brun et al. [116] found that some mutations of the viral genome can elevate the selectivity of infection, directing it to the tumor cells. In the study, MRBV was examined for its lytic properties towards human tumor cells, such as breast, colon, lung, ovarian, prostate, renal, CNS, and melanoma cancer cells. The virus was highly effective in destroying cancerous cells: all tested tumor cell lines were susceptible to the cytolytic activity. In addition, MRBV was characterized by rapid virion production and high viral burst size. The results have encouraged the researchers to produce mutants with increased selectivity towards cancer cells. Double amino acid substitution (Q242R within glycoprotein and L123W in matrix protein) in the MG1 mutant diminished the potency of the modified virus to destroy normal cells while maintaining hypervirulence in the cancerous ones [116]. Subsequent studies have proven the therapeutic properties of Maraba MG1 virus in sarcoma treatment [117], human papillomavirus-positive (HPV+) malignancies [118], and ovarian cancer [119,120]. Apart from performing its direct oncolytic activity, the MG1 mutant exerts its therapeutic effect by modifying the tumor microenvironment (TME) and sensitizing it to the mechanism of the host immune response [121].

Because of MRBV’s anti-tumor potency, the molecular framework of viral infection requires closer attention and deeper investigation. An interaction between virus and host cells is reflected in the systemic scale of oncogenesis, therefore, it should be well characterized. To date, Hassanzadeh et al. [113] have conceptualized the influence of Maraba MG1 virus on the expression of Blc-xL protein. In infected cells, two factors, 4E-BP1 and eIF2α, were involved in the downregulation of global protein synthesis. However, the whole-cell translational arrest was contrasted with a substantial elevation of Bcl-xL protein levels. A study on the U2OS cells has indicated the intensification of the *Bcl-xL* mRNA translation process, probably in an eIF2α-dependent manner. Upregulation of Bcl-xL increased cell viability and consequently made it very potent to survival during infection. By counteracting the induction of the mitochondrion-dependent apoptosis pathway, it promoted the propagation of MRBV. The study on the role of Bcl-xL in viral infection control has enriched the view of intracellular mechanisms of host-virus interactions. The results have shown that Bcl-xL may be a highly important protein in viral anti-tumor therapies [113].

## 10. The Role of Bcl-xL in Schmallenberg Virus Infection

Schmallenberg virus (SBV) is a member of the *Peribunyaviridae* family and the *Orthobunyavirus* genus and contains a negative-sense ssRNA genome divided into three segments: large (L), medium (M), and small (S) ones, enclosed in an enveloped virion of 100 nm in diameter. The vRdRp is derived from the L-segment of SBV RNA. The M segment encodes a polyprotein that further undergoes proteolytic cleavage, splitting into two glycoproteins (Gn and Gc) and a nonstructural protein, NSm. The products of S-segment expression are a nucleocapsid protein (N) and nonstructural protein (NS) that are derived from the two overlapping open reading frames. SBV infects ruminants and causes hyperthermia and diarrhea and decreases milk supply. Symptoms of the infection include, but are not limited to, abortions and congenital anomalies such as hydranencephaly, scoliosis, brachygnathia, hydrocephalus, and arthrogryposis [122,123,124].

The molecular aspects of SBV infection are still not well known. The most important question is its influence on infected cell viability. Aksoy and Azkur [123] have studied the impact of SBV on the apoptotic potential of Vero cells. The research revealed that SBV infection triggered the intrinsic and extrinsic apoptosis pathways. The host cell–virus interaction resulted in the activation of both caspase-9 and caspase-8. The analyses showed that the anti-apoptotic members of the Bcl-2 family, Bcl-xL and Bcl-2, were downregulated. The outcome suggested that, to some extent, the pro-apoptotic effect of SBV infection may be due to the change in the levels of Bcl-xL. Nevertheless, further studies are necessary to investigate the importance of Bcl-xL in SBV infection and pathogenesis [123].

## 11. The Role of Bcl-xL in CoV Infection 

Coronaviruses (CoVs) are a group of viruses belonging to the *Coronaviridae* family [125] and the *Orthocoronavirinae* subfamily [126]. The CoV genome has the form of positive-sense ssRNA [125] and is encased in an enveloped helical nucleocapsid. The virion is approximately 125 nm in diameter [126]. Severe acute respiratory syndrome (SARS)coronavirus (SARS-CoV) and SARS-CoV-2 are two highly pathogenic groups of CoV. SARS-CoV is characterized by the ability to cause SARS in infected humans. An outbreak of the disease emerged in 2002 and ceased in 2003. SARS-CoV-2 is the etiological agent of coronavirus disease 2019 (COVID-19) that has not yet been eradicated [127]. On 11 March 2020, the World Health Organization labeled its outbreak a pandemic, and the status of the disease has not changed since then [128].

An early report of Yang et al. [129] revealed the possible role of Bcl-xL in the pathogenesis of SARS. A feature of the disease is the substantial loss of host lymphocytes that leads to lymphopenia, therefore, the team used Jurkat T cells to examine the pro-apoptotic ability of SARS-CoV. The cells were transfected with cDNA encoding viral envelope (E) protein. SARS-CoV E protein increased the level of apoptosis, indicating the possible reason for the SARS-associated lymphopenia. Other tests revealed that Bcl-xL overexpression prevented the SARS-CoV E-induced death of Jurkat T cells (co-transfected with both SARS-CoV E and Bcl-xL cDNA). Further experiments showed that SARS-CoV E was able to interact with Bcl-xL through the BH3-like motif of SARS-CoV E and the BH3 domain of Bcl-xL [129]

Recent bioinformatic analysis of Navratil et al. [130] revealed structural homology between the C-terminal BH3-like motifs of SARS-CoV E and SARS-CoV-2 E. It suggested that SARS-CoV-2 E protein may also target Bcl-xL to affect the viability of infected cells [130].

## 12. Conclusions

Bcl-xL plays an important role in scenarios of various viral infections. SBV has been reported to cause a decrease in the intracellular level of Bcl-xL, whereas HBV, IAV, MRBV, EBV, and HTLV-1 are responsible for its overexpression. HCV and HIV infection can exert either a stimulatory or suppressive influence on Bcl-xL expression, according to the circumstances. Overexpression of Bcl-xL may counteract apoptosis induced by the envelope protein of SARS-CoV, while downregulation of Bcl-xL may be a molecular event leading to the development of viral pathogenesis, such as HIV-driven immune impairment or HCV-associated liver disease. On the other hand, the anti-apoptotic activity of Bcl-xL can also promote viral infections. By prolonging the survival of infected cells, Bcl-xL may favor the replication of IAV and MRBV. Bcl-xL overexpression is co-responsible for the persistence of HIV infection in immune cells acting as a viral “refuge”. Bcl-xL-dependent cell resistance to pro-apoptotic stimuli can also contribute to HBV-, HCV-, EBV-, and HTLV-1-associated tumorigenesis. Moreover, Bcl-xL may promote HCV replication by affecting the cytosolic level of Ca^2+^ in cooperation with HBx. Therefore, Bcl-xL is proposed by many reports to be a promising target molecule in anti-viral treatment.

## Data Availability

Not applicable.

## Abbreviations

AIDS	acquired immunodeficiency syndrome
ANT Apaf-1	adenine nucleotide translocator apoptotic protease activating factor-1
ATLL	adult T cell leukemia/lymphoma
Bcl-xL	Bcl-extra-large
BPR	benzodiazepine peripheral receptor
CAEBV	chronic active EBV
CNS CoV	central nervous system coronavirus
ds-DNA Nef	double-stranded DNA negative regulating factor
EBV	Epstein–Barr virus
ENKTL Fas FasL	extranodal natural killer (NK)/T cell lymphoma first apoptosis signal first apoptosis signal (Fas) ligand
HA	hemagglutinin
HAM	HTLV-1-associated myelopathy
HBcAg	HBV core antigen
HBeAg	HBV e antigen
HBsAg	HBV surface antigen
HBx	HBV x protein
HCV ssRNA	hepatitis C virus single-stranded RNA
hFLSCs	human fetal liver stem cells
HIV	human immunodeficiency virus
HPV+ HBV	human papillomavirus-positive hepatitis B virus
HTLV-1	human T-lymphotropic virus type-1
IAV	influenza A virus
IFN	interferon
IMM	inner mitochondrial membrane
IMS	intermembrane mitochondrial space
JAK	Janus kinase
LMP-2A	latent membrane protein-2A
MAPK	mitogen-activated protein kinase
MPT	mitochondrial permeability transition
MRBV	Maraba virus
NA	neuraminidase
NF-ĸB	nuclear factor-ĸB
NPC	nasopharyngeal carcinoma
NS	non-structural protein
OMM	outer mitochondrial membrane
PKC	protein kinase C
pol	polymerase
PTP	permeability transition pores
RPE SARS SARS-CoV	retinal pigment epithelium severe acute respiratory syndrome severe acute respiratory syndrome (SARS) coronavirus
SBV	Schmallenberg virus
shRNA	short hairpin RNA
STAT	signal transducer and activator of transcription
SYK	spleen tyrosine kinase
Tax	transactivator from the X-gene region
TME TNF TNFSF	tumor microenvironment tumor necrosis factor tumor necrosis factor (TNF) superfamily
TSP	tropical spastic paraparesis
UACA	uveal autoantigen with coiled-coil domains and ankyrin repeats
VDAC	voltage-dependent anion channel
Vpr	viral protein R
vRdRp	viral RNA-dependent RNA polymerase
vRNP	viral ribonucleoprotein
VSV	vesicular stomatitis virus
ΔΨm	mitochondrial membrane potential
